# Trochanteric Bolt Failure in a Modular Femoral Revision System

**DOI:** 10.5435/JAAOSGlobal-D-23-00066

**Published:** 2023-09-12

**Authors:** Daniel Acevedo, Justin E. Trapana, David Constantinescu, Jaime Alberto Carvajal Alba

**Affiliations:** From the Nova Southeastern University Dr. Kiran C. Patel College of Allopathic Medicine (NSU MD), Fort Lauderdale, FL (Acevedo), and the Department of Orthopedic Surgery, University of Miami Hospital, Miami, FL (Dr. Trapana, Dr. Constantinescu, Dr. Carvajal Alba).

## Abstract

With the incidence of primary total hip arthroplasty continuing to rise in the United States, the innovation behind improving current total hip arthroplasty systems inevitably grows with it—each new design potentially ushering in new flaws. We report a case of screw failure with the Arcos Modular Femoral Revision System–Trochanteric Bolt and Claw Technique in a 74-year-old male patient. The patient presented to the investigator's clinic for their 20-month follow-up evaluation of their complex right hip revision. Radiographs revealed failure of the screw attaching the claw plate to the stem resulting in dislodgement and relocation of the screw within the intra-articular cavity. The patient elected for nonsurgical management and will continue to be monitored. Consent by the patient involved in this case report was obtained.

Total hip arthroplasty (THA) in the setting of fracture increases the risk of instability and implant subsidence.^1^ Complex periprosthetic fracture cases may require fixation of the greater trochanter—failure of which to fixate can result in bursitis, altered gait, and increased dislocation rates.^2,3,4,5^ Claw plates with multifilament cables and screws can be used to decrease the nonunion rate.^6^ However, they have been associated with high rates of revision surgery, nonunion, and implant failure.^7–9^ In this report, we present a case of implant failure in the Zimmer Biomet Arcos Modular Femoral Revision System–Trochanteric Bolt and Claw Technique.

## Case History

**Figure 1:**
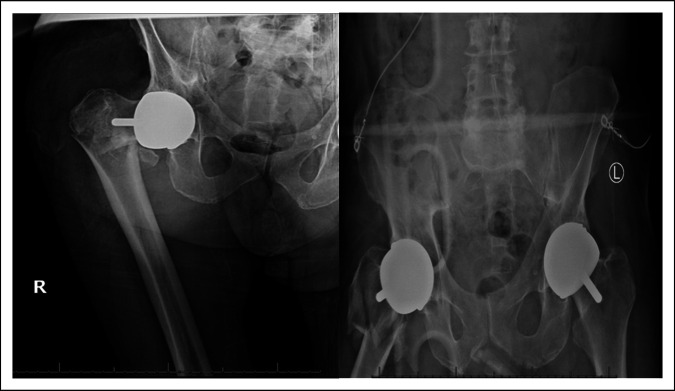
Preoperative radiographs on April 05, 2021.

The patient was a 74-year-old male with a BMI of 27.32 kg/m^2^ who underwent revision of a previous right hip resurfacing to a right total hip arthroplasty on 4/6/2021 because of a right intertrochanteric hip fracture with subtrochanteric extension. Pre-operative radiographs (04/05/2021) in figure 1, depict previous bilateral Birmingham hip resurfacing which was done more than 10 years ago at an outside facility. The revision implants used on 4/6/2021 included a Biomet Arcos modular revision hip stem 14 mm × 150 mm, Arcos standard cone proximal body size a 70 mm, dual mobility bearing 28 mm ceramic head with 52 mm outer, -6 neck length taper sleeve, 38 mm trochanteric bolt, large trochanteric claw plate, and 2 × 1.8 mm cables.

During surgery, a notable fracture hematoma was encountered, as well as comminution of the proximal femur. The dissection was carried down to the fracture; scar tissue around the acetabular shell and neck was removed. Retractors protected soft tissues while an oscillating saw and reciprocating saw were used to make a femoral neck cut. The resurfacing implant and proximal femoral bone were removed because of lack of soft-tissue connections. The acetabular shell was thought to be stable. Attention was turned to the femur; dissection was carried down to the posterior aspect; and the gluteus maximus was released. A prophylactic cable was placed around the proximal segment of the femoral canal. The distal femoral segment was presented and the canal prepped starting with the canal finder. It was sequentially reamed using a 14-mm reamer. A final 14 mm × 150 mm stem was impacted. After dual-mobility trials, we found a 70-mm proximal body to be at an appropriate leg length and were able to reduce the greater trochanteric fracture piece over the proximal body easily.

The hip was reduced and the greater trochanter fracture piece was positioned over the final body, held in place by the hook plate and jig. Instructions per technique guide for plate fixation were abided by. We then drilled a 38-mm deep screw hole for the trochanteric bolt, and the bolt was placed and secured through the large hook plate using the torque-limiting screwdriver from the device manufacturer. A distal cable was placed around the hook plate and femur.

**Figure 2:**
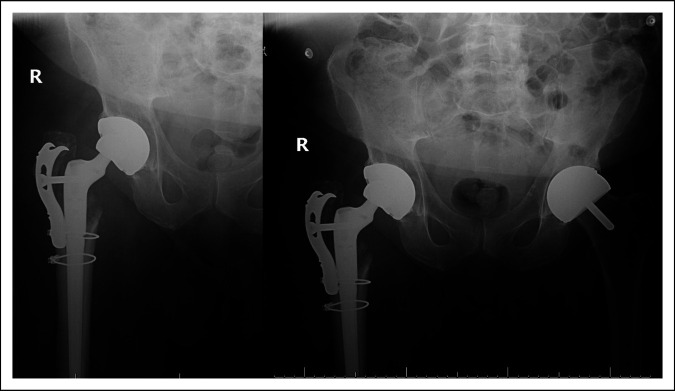
Preoperative second revision radiographs on May 09, 2021.

After the revision on April 06, 2021, the patient suffered two dislocations, both within 1 month, which required sedation and closed reduction in the Emergency Department at an outside facility. Given the patient's notable pain, disability, and clear instability, a second revision surgery was performed at that time (May 11, 2021) to replace the acetabular shell. Preoperative radiographs from 2 days before the second revision surgery (May 09, 2021) are presented in Figure 2. During the second revision, the femoral implant appeared well-fixed and in an adequate position, but the greater trochanter and gluteal attachments were not engaged with the trochanteric plate. The trochanteric fragments were repositioned and secured to the plate. The trochanteric bolt was reinserted without difficulties using the torque-limiting screwdriver once again. The repair was also reinforced using FiberWire suture around the superior aspect of the greater trochanter and the proximal body of the stem. Any soft-tissue releases were repaired under appropriate tensions. The patient tolerated both procedures well; there were no intraoperative complications; and leg-length assessment revealed that the legs were even after both procedures.

**Figure 3:**
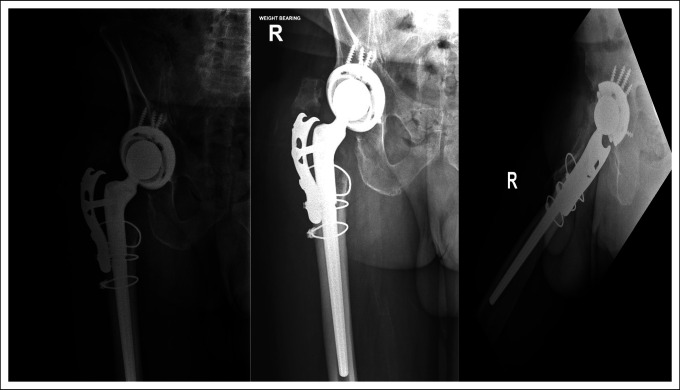
Radiographs from the 1-month follow-up on June 04, 2021.

**Figure 4:**
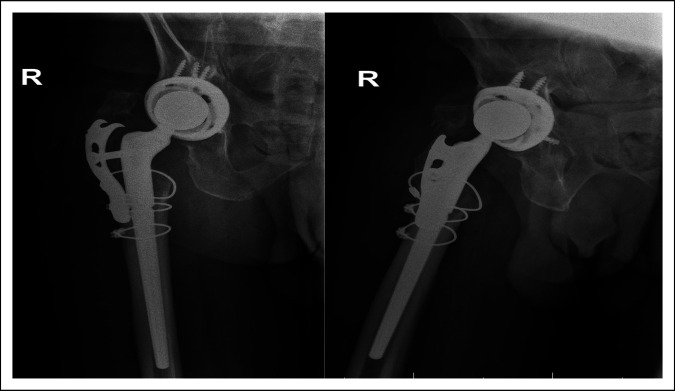
Radiographs from the 3-month follow-up on August 20, 2021.

**Figure 5:**
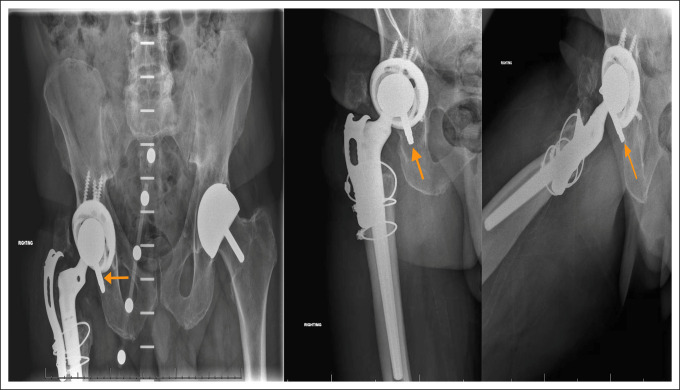
Radiographs from the 20-month follow-up on January 27, 2023.

Postoperative radiographs at 1 month, 3 months, and 20 months from the second revision are presented in Figures 3, 4, and 5, respectively.

Radiographs from the 1-month and 3-month follow-ups (Figures 3 and 4, respectively) reveal stable appearance of the right hip arthroplasty with intact implants. Radiographs from the 20-month follow-up (Figure 5) reveal that a hip arthroplasty screw (orange arrow) from the fixing plate has backed out and is currently located in the joint.

Examination of the right hip revealed a healthy-appearing surgical wound, no external signs of infection, or no signs of notable swelling. The patient was able to abduct and adduct against resistance. Sensation was grossly intact in the entire affected lower extremity. No perceived leg-length discrepancy was noted.

Despite the incidental finding of implant failure with the intra-articular location of the trochanteric bolt, the patient reported being satisfied with the surgical outcome and had no concerns during the follow-up visit. Given their presentation, the patient did not wish to proceed with a revision surgery at the time. The patient's care plan consists of routine radiograph surveillance and physical therapy.

## Discussion

Use of THA in the setting of fracture allows early ambulation and reduces bed rest complications.^10–12^ However, fixation of the greater trochanter after osteotomy can be difficult, with current literature showing no consensus regarding the best practice; the potential nonunion would require revision surgery.^9,11,13,14,15,16^ With the most common reasons for THA revision being dislocation and mechanical loosening,^6^ the Arcos Modular Femoral Revision System offers a wide-ranging, press-fit revision stem design. While it is widely used for revision surgeries, there is currently little literature regarding it. Notably, Dyreborg et al found no cases of aseptic loosening among 46 patients at a 5-year follow-up; however, they did not specify the use of the Trochanteric Bolt and Claw Technique used in this case.^11,17–19^

Possible technical errors that may have led to screw failure include a possible mismatch of the jig (trochanteric bolt guide) causing the screw to not be tightened all the way and possible backing out of the screw as a result of not being tightened all the way. There is also the possibility of an incorrectly measured screw, causing the far-sided threads to not fully engage the femoral prosthesis.

Another potential cause of screw failure includes nonunion of the greater trochanter. However, this seems unlikely in our patient who is clinically pain-free and whose radiographs have evidence of union.

Screw failures, such as in this case, compromise the increased stability and fixation that the surgeon anticipated the patient needed. Care has to be taken when removing and reinserting the screw through the stem because damage of the threads of the screw of the locking mechanism may result in bolt dislodgement. Ultimately, the patient is now at a greater risk of implant failure and metallosis.^20–22^ Revision surgery should be considered based on pain, range of motion, and functional limitations. To the best of the author's knowledge, there have not been other case reports with this issue.

## Summary

To the best of the author's knowledge, this is the first case report of in vivo trochanteric bolt failure with the Arcos Modular Femoral Revision System–Trochanteric Bolt and Claw Technique.
